# Two hemi-purse-string suture for dodenal stump reinforcement during laparoscopic gastrectomy for gastric cancer

**DOI:** 10.1186/s12957-026-04325-3

**Published:** 2026-04-14

**Authors:** Xiaodong Wang, Xinhua Chen, Tian Lin, Jingtong Lin, Danyue Deng, Xingdi Zhang, Zhishuo Li, Hao Liu, Yanfeng Hu, Jiang Yu, Mingli Zhao

**Affiliations:** 1 Department of General Surgery, Southern Medical University Nanfang Hospital, Guangzhou, China; 2 The First Clinical Medical School, Southern Medical University, Guangzhou, Guangdong 510515 China; 3 Guangdong Provincial Key Laboratory of Precision and Minimally Invasive Diagnosis and Treatment for Gastrointestinal Tumors, Guangzhou, Guangdong 510515 China

**Keywords:** Two hemi-purse-string suture, Duodenal stump reinforcement, Laparoscopic radical gastrectomy, Gastric cancer, Duodenal stump leakage

## Abstract

**Background:**

We present a novel two hemi-purse-string suture technique for duodenal stump reinforcement in laparoscopic gastrectomy. However, the simplicity and safety of this technique have not yet been fully evaluated.

**Methods:**

Retrospectively collected data from 217 patients diagnosed with gastric cancer and underwent distal/total gastrectomy at Nanfang Hospital from April 2022 to April 2025, including 107 cases underwent the two hemi-purse-string suture and 110 cases underwent continuous inverting suture. Analyze and compare the clinicopathological information, intraoperative operation time and postoperative complications between two groups of patients.

**Results:**

There was no statistically significant difference in baseline data between the two groups (*P* > 0.05), indicating comparability. Compared to the continuous inverting suture group, patients who underwent duodenal stump reinforcement with the two hemi-purse-string technique demonstrated a significantly shorter duodenal stump suture time (298.2 ± 53.8 s vs. 326.8 ± 78.4 s, t = -3.109, *p* = 0.002) and required fewer stitches (4.47 ± 0.63 vs. 6.35 ± 1.37, t = -12.892, *p* < 0.001). Regarding the success of embedding, a successful primary embedding was achieved in 100% (107/107) of cases in the two hemi-purse-string suture group, compared to 99.1% (109/110) in the continuous inverting suture group. No cases in either group experienced duodenal serosal tearing during the reinforcement procedure. The duodenal stump embedding was completed according to the predetermined plan in all patients in both groups (107/107 vs. 110/110). Importantly, no incidents of duodenal stump leakage, pancreatic fistula, or intra-abdominal haemorrhage occurred in either group, indicating comparable safety profiles between the two techniques.

**Conclusion:**

The two hemi-purse-string suture used for duodenal stump reinforcement can shorten the suture time and has no postoperative complications such as duodenal stump fistula. It is efficient and reliable.

**Supplementary Information:**

The online version contains supplementary material available at 10.1186/s12957-026-04325-3.

## Introduction

Gastric cancer (GC) ranks among the most prevalent solid tumors worldwide, exhibiting both high incidence and mortality rates that place it as a main cause of cancer-related deaths [1]. Despite considerable advancements in diagnostic modalities and therapeutic strategies over recent decades, radical gastrectomy remains the primary curative intervention for patients with early-stage and locally advanced gastric cancer [2]. 

Duodenal stump leakage (DSL) represents a rare yet potentially catastrophic complication following gastrectomy, associated with severe sequelae including intra-abdominal abscess, sepsis, and multi-organ dysfunction syndrome (MODS), which collectively contribute to substantial morbidity and mortality. The clinical necessity of duodenal stump reinforcement (DSR) during gastrectomy remains contentious within the surgical community. While some meta-analyses argue that DSR fails to significantly reduce DSL incidence [3], mounting evidence from cohort studies suggests that selective reinforcement may mitigate this risk, particularly in high-risk patient populations or technically challenging dissections [4, 5]. Further research into its pathophysiological mechanisms reveals that impaired local microcirculation (insufficient blood supply) and mechanical tension imbalance at the closure interface are core factors contributing to DSL. Reinforcement techniques such as seromuscular suturing or vascularized omental coverage can significantly reduce the incidence of DSL by improving stump perfusion and enhancing mechanical stability. The international consensus on standardization of data collection for complications associated with esophagectomy, jointly developed by multiple international societies, concluded that failure to reinforce the duodenal stump is a modifiable risk factor for DSL (evidence level ⅡB) [6]. Particularly in totally laparoscopic surgery, this consensus globally incorporated duodenal stump reinforcement into the standardized procedural steps for gastric cancer surgery for the first time.

Laparoscopic gastrectomy has been widely recognized as a minimally invasive yet technically demanding procedure that currently represents the standard of care for distal and total gastrectomy according to major clinical guidelines. While offering advantages over open approaches, laparoscopic duodenal stump reinforcement presents unique technical challenges that demand advanced surgical expertise. Although several reinforcement techniques have been reported in the literature, optimal approaches remain debated. Herein, we introduce and describe a novel reinforcement technique - the two hemi-purse-string suture method - for duodenal stump reinforcement during laparoscopic gastrectomy. 

## Methods

### Statement

This study involves medical data from human subjects. All procedures in this study comply with the World Medical Association’s Declaration of Helsinki (2013 version). The study has been approved by the Ethics Review Committee of Nanfang Hospital, Southern Medical University (approval number: NFEC-2025-565). Informed consent was obtained from all individual participants included in the study. 

### Study design

**Fig. 1:**
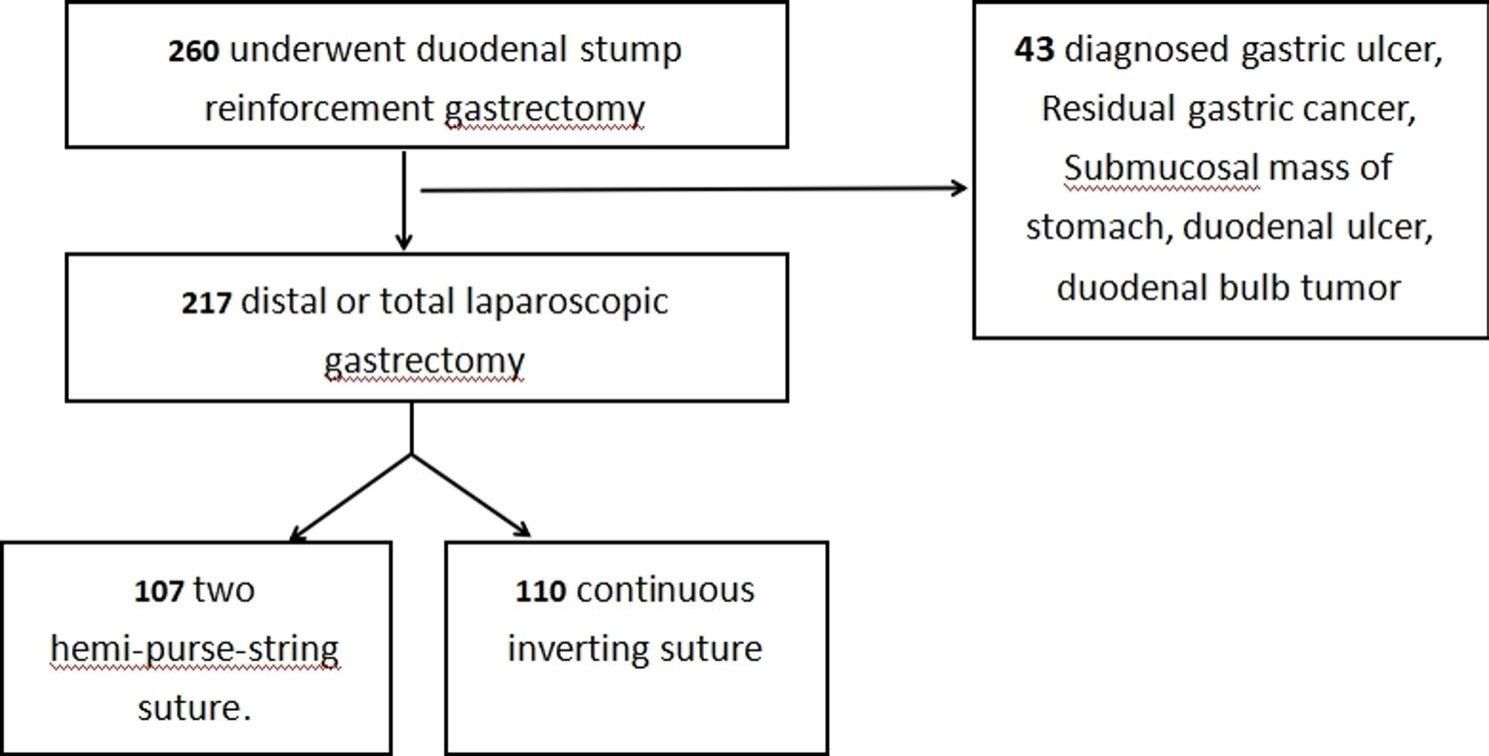
Patient selection flowchart

We retrospectively analyzed clinical data from 217 consecutive patients who underwent either distal or total laparoscopic gastrectomy with intracorporeal duodenal stump reinforcement at Nanfang Hospital, Southern Medical University between April 2022 and April 2025. The cohort was divided into two groups based on the reinforcement technique: 107 patients received the two hemi-purse-string suture technique, while 110 patients underwent continuous inverting suture. The patient screening flowchart is presented in Fig. 1. All surgeries were performed by senior attending surgeons. Cases from the surgeons’ learning curve—specifically, the first 20 laparoscopic procedures performed using each reinforcement technique—were excluded to minimize the impact of technical instability on the outcomes. The choice of reinforcement method was based on the surgeon’s clinical experience and individualized patient assessment. Data on clinicopathological characteristics, surgical outcomes, and postoperative recovery were collected from the electronic medical record system. The reinforcement method and suturing time were recorded based on surgical video reviews, with the operation time defined as the interval from the first suture placement to the completion of the final knot.

#### Inclusion criteria

Consecutive adult patients (≥ 18 years) who underwent laparoscopic radical gastrectomy with duodenal stump reinforcement between April 2022 and April 2025 were included. Gastrointestinal reconstruction methods included Bilroth II and Roux-en-Y anastomosis.

#### Exclusion criteria

- Diagnosis of gastric ulcer, remnant gastric cancer, gastric submucosal tumor, or duodenal bulb tumor;
- Emergency surgery or palliative surgery;

#### Handling of missing data

A total of 217 patients were included in this study. All data on preoperative indicators, surgical details, and postoperative complications were complete and available for all cases, with no missing values. Consequently, no processing for missing data was performed.

### Surgical procedure

All procedures were performed by board-certified chief surgeons with specialized expertise in laparoscopic gastrointestinal surgery. Gastrectomy with D2 lymphadenectomy was performed in both groups using the technique detailed in our previous study [7–11]. The resected stomach and lymph nodes were extracted through the extended umbilical incision, assessed to ensure free resection margins, and then routinely processed for further examination [12].Consistent with open surgical standards, the extent of resection in laparoscopic gastrectomy is primarily determined by tumor location in strict accordance with the Japanese Gastric Cancer Association (JGCA) treatment guidelines (5th edition) [13]. Following complete lymph node dissection, duodenal transection was achieved using a 60-mm endoscopic linear stapler positioned 2–3 cm distal to the pyloric ring. Subsequent duodenal stump reinforcement was systematically performed using a 3 − 0 STRATAFIX™ Spiral PGA-PCL knotless barbed suture (model SXMD1B405, Ethicon Inc., Somerville, NJ, USA) in continuous fashion, with particular attention paid to ensuring seromuscular layer incorporation and adequate tissue approximation. 

### Duodenal stump reinforcing method

#### Continuous inverting suture

Perform 5 to 6 continuous mattress inversion sutures and then tighten the sutures to embed the duodenal stump [14]. 

#### Two hemi-purse-string suture

Video 1 shows the Two hemi-purse-string suture.

##### Step 1

**Fig. 2:**
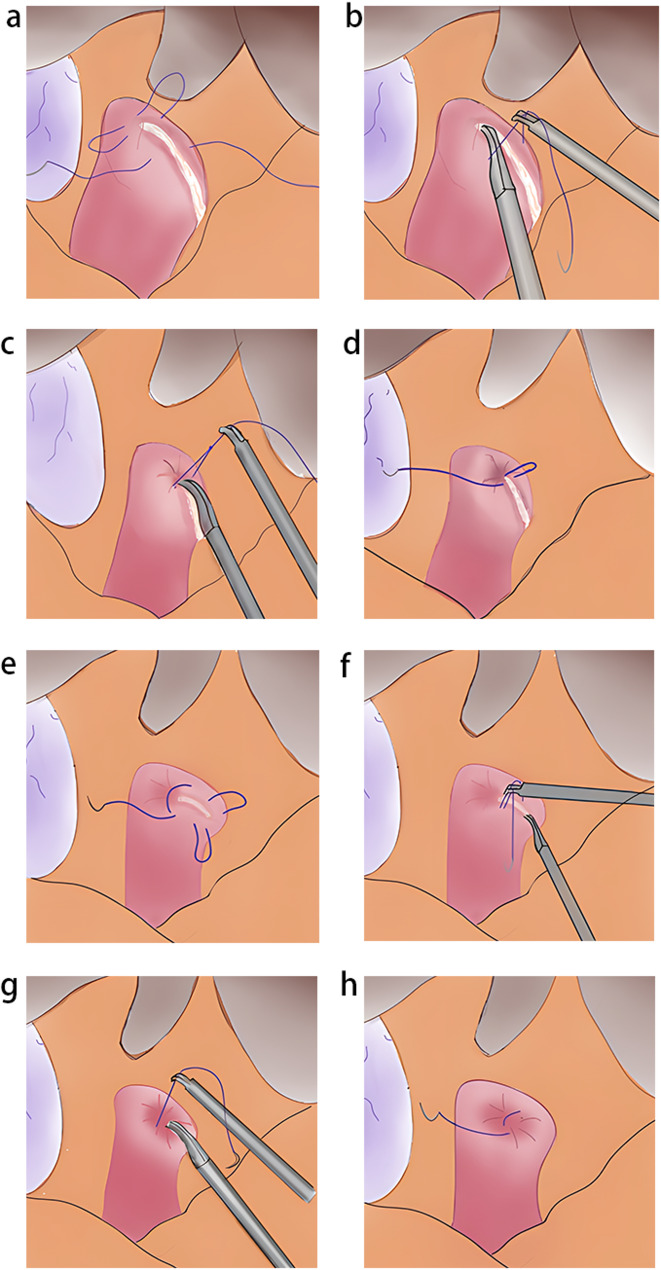
Schematic picture of two hemi-purse-string suture

**Fig. 3:**
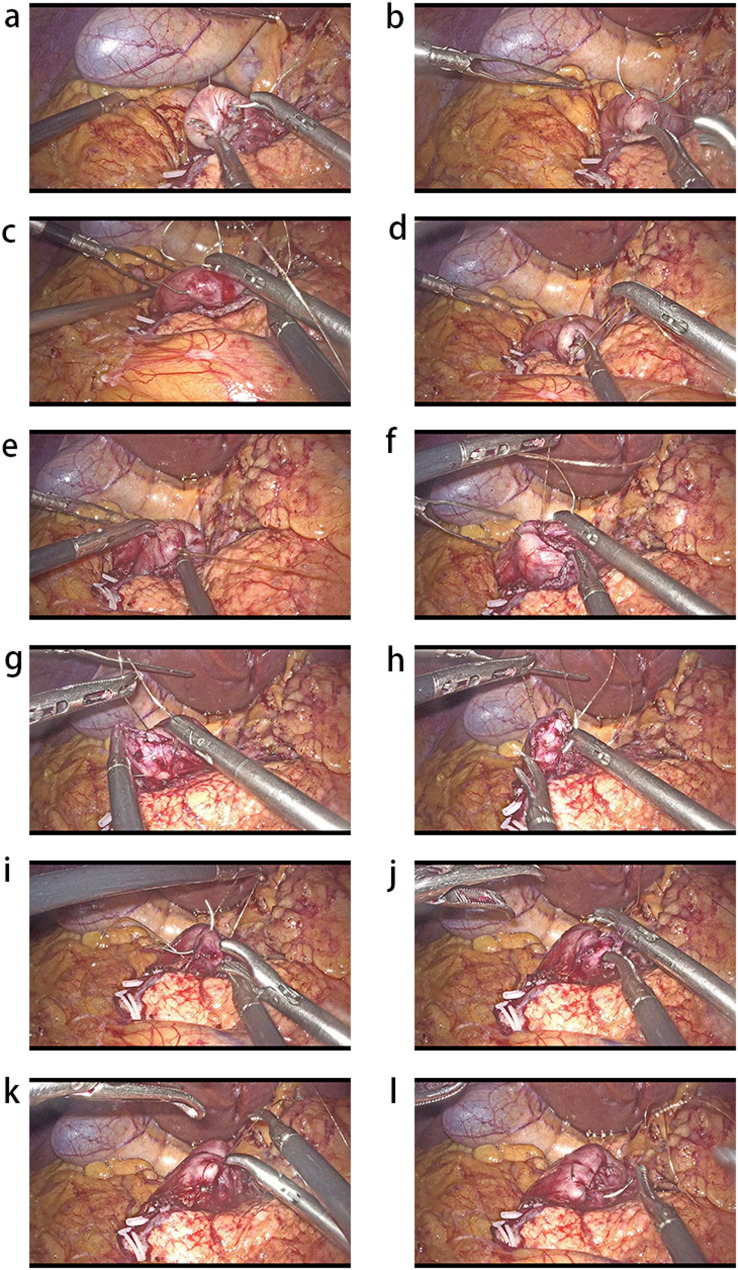
Physical picture of two hemi-purse-string suture

The first stitch is placed approximately 1.0–1.5 cm from the closure line, slightly above its midpoint. Suturing is then performed counterclockwise at the left, upper, and right aspects of the duodenal stump (Figs. 2a and 3a-c). After passing the suture through the knotting hole to form a loop, the knot is not tightened immediately. The surgeon holds both the left and right ends of the suture near the knotting hole with a needle holder in the right hand, while grasping the upper corner of the stump with a dissecting forceps in the left hand (Figs. 2b and 3d). The stump is inverted into the purse-string, after which the suture above the knotting hole is grasped and tightened to complete the upper hemi-purse-string (Figs. 2c and 3e).

##### Step 2

A single stitch is placed at the opening of the upper hemi-purse-string to reinforce it (Figs. 2d and 3f).

##### Step 3

Similar to the first step, a stitch is placed approximately 1.0–1.5 cm from the closure line, slightly below its midpoint. Suturing is then performed clockwise at the left, lower, and right aspects of the duodenal stump (Figs. 2e and 3g-i). The surgeon holds both the left and right ends of the suture near the knotting hole with a needle holder in the right hand, while grasping the lower corner of the stump with a dissecting forceps (Figs. 2f and 3j). The stump is inverted into the purse-string, and the suture above the knotting hole is then tightened to complete the lower hemi-purse-string (Figs. 2g and 3k).

##### Step 4

A final reinforcing suture secures the inferior hemi-purse completion. (Figs. 2h and 3l).

### Statistical methods

All statistical analyses were performed using IBM SPSS Statistics software (version 26.0; IBM Corp.). Continuous variables were expressed as mean ± standard deviation (SD), while categorical variables were presented as frequencies and percentages. Between-group comparisons were conducted using the independent Student’s *t*-test for continuous variables and the χ² test or Fisher’s exact test for categorical variables. A two-tailed *P*-value of < 0.05 was considered statistically significant. 

### Observation period

Duodenal stump leakage (DSL) primarily occurs 2–7 days after surgery. Therefore, the main observation period in this study covered the entire postoperative hospital stay (average 7–10 days), which was sufficient to detect the majority of DSL events. We also conducted follow-up for one month after surgery to identify the very rare cases of delayed-onset DSL that might occur after discharge. 

### Complications evaluation

According to the International Consensus on Standardized Definitions for Postoperative Complications of Gastric and Esophageal Cancer, duodenal stump leakage (DSL) was defined as any of the following manifestations occurring ≥ 3 days postoperatively:

- ① Contrast extravasation from the stump confirmed by imaging (CT/contrast radiography);
- ② Bile (bilirubin > 3 times the serum level) or pus detected in abdominal drainage fluid;
- ③ Stump rupture confirmed by surgical exploration.

Severity was graded in accordance with the consensus: Grade A (asymptomatic), Grade B (requiring interventional therapy), Grade C (requiring surgical intervention).

Other complications were graded according to the Clavien–Dindo classification system.

## Results

### Patients’ clinicopathological characteristics

The study cohort comprised 217 patients, with 107 underwent duodenal stump reinforcement via the two hemi-purse-string suture technique and 110 receiving conventional continuous inverting suture. There were no statistically significant differences between the two groups of patients in terms of age, gender, BMI, presence of diabetes mellitus, preoperative albumin levels, type of gastrectomy, maximum tumor diameter, presence of signet-ring cell carcinoma, T stage, N stage, or tumor location (all *P* > 0.05, Table 1).

**Table 1:** Patients’ clinicopathological characteristics

Variables	Two hemi-Purse-String Suture (107)	Continuous inverting suture (110)	Values	*P*
Age (years, x̄ ± s)	57.5 ± 12.5	58.1 ± 12.6	t=-0.349	0.727
Sex [n(%)]			Χ²=0.013	0.910
Male	65（60.7%）	66（60.0%）		
Female	42（39.3%）	44（40.0%）		
BMI (kg/m2) [n(%)]			Χ²=1.142	0.285
＜25	78（72.9%）	87（79.1%）		
≥25	29（27.1%）	23（20.9%）		
Diabetes mellitus [n(%)]			Χ²=2.172	0.141
Yes	8（7.5%）	15（13.6%）		
No	99（92.5%）	95（86.4%）		
Albumin (g/L,x± s)	39.6 ± 4.2	39.0 ± 4.1	t=0.843	0.401
Type of gastrectomy [n(%)]			Χ²=1.007	0.316
Distal	66（46.8%）	75（68.2%）		
Total	41（53.2%）	35（31.8%）		
Maximum diameter of tumor（cm, x̄ ± s）	2.96±1.95	3.26±2.69	t=-0.919	0.359
Signet-ring cell carcinoma [n(%)]			Χ²=0.530	0.467
Yes	50（46.7%）	46（41.8%）		
No	57（53.3%）	64（58.2%）		
Pathological T stage [n(%)]			U=5516.5	0.410
(y)pT0	6（5.6%）	7（6.4%）		
(y)p T1	36（33.6%）	33（30.0%）		
(y)p T2	15（14.0%）	11（10.0%）		
(y)p T3	30（28.0%）	32（29.1%）		
(y)p T4a	18（16.8%）	27（24.5%）		
(y)p T4b	2（1.9%）	0（0%）		
Pathological N stage [n(%)]			U=5725.5	0.707
(y)p 0	55（51.4%）	61（55.5%）		
(y)p 1	16（15.0%）	12（10.9%）		
(y)p 2	10（9.3%）	9（8.2%）		
(y)p 3a	15（14.0%）	18（16.4%）		
(y)p 3b	11（10.3%)	10（9.1%)		
Tumor location [n(%)]			Χ²=2.837	0.242
Upper third of stomach	33（30.8%）	23（20.9%）		
Middle third of stomach	16（15.0%）	20（18.2%）		
Lower third of stomach	58（54.2%）	67（60.9%）		

### Comparison of operative outcomes between the two groups

The operative outcomes for both groups are summarized in Table 2. Compared to the continuous inverting suture group, patients who underwent duodenal stump reinforcement with the two hemi-purse-string technique demonstrated a significantly shorter duodenal stump suture time (298.2 ± 53.8 s vs. 326.8 ± 78.4 s, t = -3.109, *p* = 0.002) and required fewer stitches (4.47 ± 0.63 vs. 6.35 ± 1.37, t = -12.892, *p* < 0.001). Regarding the success of embedding, a successful primary embedding was achieved in 100% (107/107) of cases in the two hemi-purse-string suture group, compared to 99.1% (109/110) in the continuous inverting suture group. For case with inversion failure, re-embedding was performed, and all secondary embedding procedures were successful in this study. No cases in either group experienced duodenal serosal tearing during the reinforcement procedure. The duodenal stump embedding was completed according to the predetermined plan in all patients in both groups (107/107 vs. 110/110). Both techniques exhibited excellent safety profiles, with zero incidence of postoperative duodenal stump leakage, intra-abdominal hemorrhage, or pancreatic fistula across the study cohort.

**Table 2:** Comparison of operative outcomes between the two groups

Variables	Two hemi-Purse-String Suture (107)	Continuous inverting suture (110)	Values	*P*
Duodenal stump suture time (seconds, x̄ ± s)	298.2 ± 53.8	326.8 ± 78.4	t=-3.109	0.002
Number of stitches (x̄ ± s)	4.47 ± 0.63	6.35 ± 1.37	t=-12.892	< 0.001
Successful primary embedding [n (%)]	107(100%)	109(99.1%)	-	
Duodenal serosal tearing [n (%)]	0	0	-	
Completion of embedding according to the predetermined plan [n (%)]	107(100%)	110(110%)	-	1.000
Duodenal stump leakage [n(%)]	0	0	-	1.000
Pancreatic fistula [n(%)]	0	0	-	1.000
Intra-abdominal haemorrhage [n(%)]	0	0	-	1.000

## Discussion

This comparative study systematically evaluated the efficacy and safety of two duodenal stump reinforcement techniques: the two -hemi-string suture versus conventional continuous inverting suture. Our findings demonstrate that the two hemi-purse-string technique offers statistically significant improvements in operative efficiency while maintaining equivalent safety outcomes, with no cases of duodenal stump leakage observed in either cohort. These results suggest that this innovative reinforcement method may represent a technically superior approach for duodenal stump management during laparoscopic gastrectomy.

DSL remains one of the most severe complications following radical gastrectomy for gastric cancer, despite recent advances in surgical techniques. Although contemporary studies report a reduced incidence of 1.1%–2.5% [15]. DSL continues to pose a substantial clinical challenge due to its associated morbidity and mortality. Established patient-related risk factors for DSL include advanced age, poor nutritional status, and significant comorbidities [16]. From a technical perspective, accumulating evidence supports that intraoperative reinforcement of the duodenal stump significantly reduces DSL risk [17, 18]. Notably, even in cases where leakage occurs despite reinforcement, clinical outcomes are markedly improved compared to non-reinforced cases, with most patients achieving resolution through conservative management. This observation suggests that reinforcement not only prevents DSL but also mitigates its severity when it does occur [19].

Duodenal stump reinforcement under laparoscopy presents significantly greater technical challenges compared to open surgical techniques. Multiple duodenal stump reinforcement techniques have been developed based on surgical preferences and technical considerations, including the BPA sheet with fibrin sealant [20], Lembert suture [21], barbed suture [14, 22], and single purse-string suture [23]. While these methods are clinically feasible, each presents distinct limitations that warrant consideration. The BPA sheet with fibrin sealant offers notable technical efficiency by eliminating the need for suturing. However, safety concerns persist, as demonstrated by Paik et al.‘s reporting a 1.6% DSL incidence (5/311 cases) with one mortality [20]. Lembert sutures, while technically straightforward, may inadequately reinforce the upper and lower duodenal angles [21]. These persistent limitations continue to drive surgical innovation in duodenal stump reinforcement methods.

Our team has developed an optimized reinforcement approach: the two hemi-purse-string suture technique. The optimized method offers several advantages. Firstly, by embedding the upper and lower portion of the stump through dual half-purses, technical difficulty in stump inversion is reduced, particularly with large cross-sectional areas and significant duodenal ulcer scarring compared to the single purse-string technique [24]. Secondly, it provides superior reinforcement at both the upper and lower corners, potentially reducing postoperative leakage rates. Thirdly, this technique can be reliably performed by a single experienced surgeon without assistant dependency and eliminate the disadvantages associated with the assistant’s limitations. Fourthly, the use of knotless sutures has the advantage of eliminating the need for intraoperative knotting, significantly improving the reinforcement efficiency. Last, a particularly ingenious aspect of the method involves the surgeon’s use of a needle holder in the right hand to grasp both the left and right sides of the suture loop simultaneously, while employing a dissecting forceps to hold the stump corner. This utilizes mechanical principles to facilitate simple and rapid inversion of the stump into the purse. After the stump is tucked in, the suture above the loop is grasped and tightened to complete the lower hemi-purse-string. This meticulously designed step is crucial for the smooth and efficient execution of the entire procedure.

Compared with other reinforcement methods, the two hemi-purse-string suture has certain advantages in efficiency. In this study, the average time of this technique was 298.2 ± 53.8 s, while that of the continuous inverting suture as the control group was 326.8 ± 78.4 s, with a statistically significant difference (*P* = 0.002). In other related studies, the average time of the single purse-string suture was about 5 min, and that of the barbed suture was 8 min on average. In terms of safety, no postoperative complications such as DSL occurred in patients who underwent the two half-purse-string suture cohorts in this study.

It is important to note that while the principle of continuous seromuscular inversion for duodenal stump reinforcement is a general surgical concept, the technical execution described in this report leverages the specific properties of barbed sutures, namely its self-anchoring capacity and ability to maintain uniform tension without knot tying. Reproducibility with conventional smooth sutures may require additional technical adjustments, such as the use of locking stitches or maintaining constant assistant traction, to achieve a similar degree of tension distribution and inversion. Further comparative studies would be valuable to determine the optimal suture material for this specific application.

While this study demonstrates promising results, several limitations must be acknowledged. Firstly, as a retrospective analysis, this study is inherently subject to selection bias and potential confounding factors that may influence the interpretation of outcomes. The non-randomized nature of patient allocation limits the strength of our conclusions. Secondly, although our preliminary findings are encouraging, the relatively small cohort reduces the statistical power to detect significant differences in rare outcomes such as duodenal stump leakage. The results should therefore be interpreted with caution until validated in larger studies. To definitively compare the efficacy and safety of different duodenal stump reinforcement techniques, large-sample and multi-center randomized controlled studies are needed.

In this study, we introduce a novel two hemi-purse-string suture technique for duodenal stump reinforcement during laparoscopic gastrectomy. Our findings demonstrate that this method offers significant advantages in both operative efficiency and technical simplicity, with no observed cases of DSL or other major complications in our cohort.

## Supplementary Information

Supplementary Material 1.

## Data Availability

The datasets generated and analysed during the current study are not publicly available due to ethical restrictions but are available from the corresponding author (xinhuachen03@163.com) on reasonable request.
